# Quantifying treatment burden: the patient burden score a study of 758 patients across three clinical urologic scenarios

**DOI:** 10.1007/s00345-024-05378-3

**Published:** 2024-11-27

**Authors:** Ofer N. Gofrit, S. Nahum Goldberg, Amitay Lorber, Mordechai Duvdevani, Marc Wygoda, Guy Hidas, Vladimir Yutkin, Liat Appelbaum Pikarsky

**Affiliations:** Departments of Urology, Radiology, and Oncology, Hadassah Hebrew University Hospital, P.O.Box 12000, Jerusalem, 91120 Israel

**Keywords:** Burden of treatment, Small renal mass, Upper ureteral stone, Muscle invasive bladder cancer

## Abstract

**Objectives:**

To develop a comprehensive scale that measures the three burden types of any treatment, including expected, unexpected (complications), and need for ancillary procedures.

**Methods:**

A panel of experts created a scale that assessed the burden of all aspects of treatment, including hospitalization, anesthesia, surgery, and follow-up. The total score is defined as the burden score (BS). BS was calculated retrospectively for patients in three clinical scenarios in urology, each with two acceptable treatment options: patients with a small renal mass (T1a) treated with either partial nephrectomy (PN, 139 patients) or percutaneous ablation (PA, 83 patients), patients with bladder cancer (stages T2-4a, N0, M0) treated with radical cystectomy (RC, 162 patients) or trimodal therapy (TMT, 88 patients), and patients with upper ureteral stones ≤ 10 mm treated with either ureteroscopy (137 patients) or extracorporeal shock-wave lithotripsy (SWL, 150 patients).

**Results:**

Both PN and PA provided excellent oncological results (5-year recurrence-free survival ≥ 97%) and low complication rates. However, the BS of PN was more than twice that of PA (27.3 ± 7.7 vs. 12.5 ± 6.4, *p* < 0.01). RC and TMT showed identical 3-year disease-specific survival rates (73%), but the BS of TMT was significantly lower (53.8 ± 11.1 vs. 42.0 ± 11.6, *p* < 0.01). Both ureteroscopy and SWL have achieved high stone-free rates (≥ 97%) and low complication rates. However, the BS of ureteroscopy was significantly lower (7.8 ± 3 vs. 9.0 ± 3.5, *p* < 0.01).

**Conclusion:**

PA treatment for small renal masses, TMT for muscle-invasive bladder cancer, and ureteroscopy for upper ureteral stones provided similar success rates to those of PN, RC, and SWL, but with significantly lower BS. This tool can assist in patient consultation when multiple treatment options are available. The concept of BS can be extended to other fields of medicine.

**Supplementary Information:**

The online version contains supplementary material available at 10.1007/s00345-024-05378-3.

“Measurement is the first step that leads to control and eventually to improvement”

Dr. H. James Hartington

When choosing among different treatment options, patients are concerned with both the success rate and the expected burden of each choice. Measuring success is relatively straightforward as it involves achieving specific goals and can be assessed using parameters such as stone-free rates or 5-year disease-free survival. However, measuring the burden of treatment is more complex. It consists of the following three components:

1. Expected burdens: These include hospitalization, anesthesia, surgery, pain management, potential mutilation associated with surgery, need for follow-up imaging studies, and additional clinic visits.
2. Unexpected downsides: These include complications, such as postoperative bleeding, infection, and thromboembolic events.
3. Need for ancillary procedures: This refers to additional interventions required if the initial treatment fails, such as ureteroscopy following shockwave lithotripsy (SWL) to achieve “stone-free” status, or vice versa.

Although patients are concerned with all three burden types, the current standard scales typically measure only the second type. The modified Clavien system is the most used scale for assessing complication severity [1]. This scale evaluates the effort required to restore the pre-complication status but intentionally excludes expected mutilation sequelae of surgery, such as bladder loss after radical cystectomy and its implications for the patient. It also overlooks the burden associated with the need for ancillary procedures following an initial treatment failure. Similarly, in Oncology, the Common Terminology Criteria for Adverse Events v 4.0, and Radiation Oncology/Toxicity grading/RTOG assess complications related to chemotherapy and radiotherapy [2, 3].

Patients are clearly concerned with all three burdens. In this study, we developed a scale that combined expected downsides, unexpected complications, and the need for ancillary interventions following initial treatment failure. The goal was to provide patients, physicians, and healthcare managers with a tool to compare different treatments. This tool, in addition to success rate, can serve as an adjunct factor for choosing the most appropriate treatment when multiple treatment options are available.

## Methods

### Patient selection

The patients included in the study were treated in a tertiary center and presented with three common urologic entities for which at least two acceptable treatment options are available: (1) small renal masses (T1a, N0, M0) (2) muscle-invasive bladder cancer (T2-4a, N0, M0); (3) Upper ureteral stones ≤ 10 mm. The minimum follow-up period was six months (unless the patient died earlier). Comorbidities were determined using the Charlson Comorbidity Index (CCI) [4]. This study was approved by the local IRB (0323-24-HMO) and waived the requirement for informed consent.

### Small renal masses (stage T1a)

These tumors can be treated with percutaneous ablation (PA) or laparoscopic robot-assisted partial nephrectomy (PN) [5, 6]. PA was performed using a high-power microwave (Medtronic, Minneapolis, Minesota) or radio frequency generator (Cambridge Interventional CRF System, Burlington, MA), according to the manufacturer’s guidelines. PN was conducted using the Da Vinci X or Xi system (Intuitive^®^ Sunnyvale, California, USA). The treatment modality was chosen according to the discretion of the patient and physician. Tumor recurrence was assessed using imaging studies that identified distant metastases, a locally enhancing mass, or a locally expanding mass in patients who could not tolerate intravenous contrast injection. The monitoring protocol after treatment included imaging at 3, 6, and 12 months and annually thereafter. The patients included in the study underwent treatment between the years 2010–2022.

### Urothelial muscle invasive bladder cancer (stages T2-4a, N0, M0)

Patients with urothelial muscle-invasive bladder cancer (T2-4a, N0, M0) can be treated with either neoadjuvant chemotherapy followed by radical cystectomy (RC) or trimodal therapy, which includes maximal transurethral resection followed by chemoradiation [7]. The protocol included chemotherapy with weekly cisplatin (35–40 mg/m^2^ weekly) in eligible patients and weekly carboplatin (AUC 2) in all other patients. Intensity-modulated radiotherapy was administered using either a Varian TrueBeam Linear accelerator for classical intensity-modulated radiotherapy or an ETHOS^™^ Varian linear accelerator (Palo Alto, California) for Adaptive Radiotherapy, and included 44–46 Gy in 1.8–2 Gy fractions to the bladder/pelvic nodes followed by a boost of 16–20 Gy (in 2 Gy fractions), bringing the total dose to 60–66 Gy. RC was done openly in all patients. Patients with synchronous or metachronous upper urinary tract tumors and those with other uncontrolled malignancies were excluded. The patients included in the study underwent treatment between the years 2001–2022.

### Upper ureteral stones ≤ 10 mm

These patients can be managed with ureteroscopy or extracorporeal SWL [8]. SWL was performed using a Gemini lithotripsy unit (Dornier MedTech, Munich, Germany). Ureteroscopy was performed using a Flexible Uretero-Renoscope (KARL STORZ, Tuttlingen, Germany) and a pulse 120 H Holmium Laser System with MOSES Technology (Lumenis, Yokne’am Illit, Israel). Stone-free rate was defined as the absence of residual fragments or fragments ≤ 2 mm 1 month after surgery. Patients with more than one stone or a metabolic disorder were excluded from the trial. The patients included in the study underwent treatment between the years 2021–2022.

### Weights of the scale

The weights of the scales were determined by a multidisciplinary committee of clinicians attempting to reflect the burden of treatment on the patients. The weights were designed to encompass most aspects of therapy, including the nuisance associated with hospitalization, anesthesia, surgery, pain management, organ loss, unexpected events (complications), post-treatment follow-up, and the need for ancillary procedures (with an additional weight of two points beyond the weight of the procedure itself). A complete list of weights is provided in Supporting Information 1. The burden score (BS) was the sum of all the weight points. Disease recurrence was not considered a burden and was evaluated separately.

### Statistics

All analyses were based on the intention-to-treat principle. All complications and outcomes were assessed during the final follow-up visit. Continuous variables were compared using an unpaired t-test and categorical values were compared using Fisher’s exact test. Survival was analyzed using the Kaplan–Meier method with a log-rank test. Statistical significance was set at p value < 0.05.

## Results

### Patients with small renal mass (stage T1a)

A total of 83 patients underwent PA and 139 underwent PN for a solitary T1a kidney tumor. Their basic parameters and outcomes are presented in Table 1. The complete dataset is provided in Supporting Information 2. Patients treated with PA were significantly older, had a significantly higher CCI, and their tumors were smaller by an average of 6 mm than those treated with PN. Both PN and PA were highly successful, with a 5-year recurrence-free survival of ≥ 97%. However, 11 patients treated with PA required an ancillary procedure (including nine repeated PAs and two PNs) compared to a single patient who underwent a second PN. Complications were rare in both groups. The average BS of patients undergoing PA was less than half of that of PN (12.5 ± 6.4 vs. 27.3 ± 7.7, *p* < 0.01). The BS of uncomplicated PA, including daycare hospitalization, anesthesia by mask, and oral analgesics was to 9.5–10, compared to 24 in uncomplicated PN. When more than one PA session was necessary, the BS was 18–20, still lower than a single PN.

**Table 1:** Basic parameters, with outcome and burden scores, of patients with small renal mass treated with either percutaneous ablation or partial nephrectomy

	Percutaneous ablation	Partial nephrectomy	*p* value
Number of patients	83	139	
Average age ± SD	68.0 ± 10.0	58.5 ± 13.2	< 0.01
Average Charlson comorbidity index ± SD	1.1 ± 1.7	0.31 ± 0.82	< 0.01
Average largest diameter ± SD (range) cm	2.2 ± 0.66 (0.9–3.8)	2.8 ± 0.75 (1.2-4)	< 0.01
Median follow-up (Q1, Q3) months	36.0 (19.0,68.0)	31.5 (17.0,51.8)	0.058
Five-year recurrences-free survival	100%*	97.9%*	0.53
Average burden score ± SD	12.5 ± 6.4	27.3 ± 7.7	< 0.01

*Eleven patients (13.2%) initially treated with PA required an ancillary procedure to achieve complete treatment compared to a single patient (0.7%) after PN

### Patients with muscle invasive bladder cancer (stages T2-4a, N0, M0)

A total of 162 patients underwent RC, and 88 underwent TMT for T2-4a, N0, and M0 bladder cancer. Their basic parameters and outcomes are presented in Table 2, and the complete dataset is provided in Supporting Information 3. Patients treated with TMT were significantly older than those treated with RC; however, both groups had a similar CCI. Follow-up duration and disease-specific survival rates were similar. Twelve patients who were initially treated with TMT underwent salvage RC. The average BS of patients undergoing TMT was about 25% lower than that of RC (53.8 ± 11.1 vs. 42.0 ± 11.6, *p* < 0.01). The BS of a typical uncomplicated TMT was approximately 40, and that of an uncomplicated RC was 55 with ileal conduit reconstruction and 45 with continent diversion.

**Table 2:** Basic parameters, with outcome and burden scores, of patients with T2-4a, N0, M0 bladder cancer treated with either radical cystectomy or trimodal therapy

	Radical cystectomy	Trimodal therapy	*p* value
Number of patients	162	88	
Average Age ± SD	70.7 ± 10.1	75.7.5 ± 10.7	< 0.01
Average Charlson Comorbidity Index ± SD	0.41 ± 0.73	1.68 ± 1.52	0.71
Median follow-up (Q1, Q3) months	32.0 (11.0,68.0)	31.5 (17.0,51.8)	0.25
Three-year disease-specific survival	73.5%*	73.2%	0.35
Average burden score ± SD	53.8 ± 11.1	42.0 ± 11.6	< 0.01

*Twelve patients (13.6%) required RC after TMT

### Patients with upper ureteral stones (≤ 10 mm)

A total of 137 patients underwent ureteroscopy and 150 underwent SWL for upper ureteral stones ≤ 10 mm (Table 3). The complete dataset is provided in Supporting Information 4. The groups were similar in terms of stone diameter (average, 7.3 mm in both groups); however, patients treated with SWL were significantly younger. Both modalities provided high stone-free (≥ 97%) and low complication rates; however, 16% of the patients treated with SWL required a secondary procedure to achieve this rate, compared with 5.1% of those who underwent ureteroscopy (*p* < 0.01). The BS of ureteroscopy was significantly lower than that of SWL (7.8 ± 3.0 vs. 9.0 ± 3.5, *p* < 0.01).

**Table 3:** Basic parameters, with outcome and burden scores, of patients with upper ureteral stone ≤ 10 mm treated with either ureteroscopy or shockwave lithotripsy

	Ureteroscopy	Shockwave lithotripsy	*p* value
Number of patients	137	150	
Average Age ± SD	51.2 ± 14.1	45.1 ± 13.3	< 0.01
Average Charlson Comorbidity Index	0.18 ± 0.65	0.07 ± 0.29	0.07
Average largest stone diameter (mm)	7.3 ± 1.6	7.3 ± 1.8	0.874
Secondary procedure	7 (5.1%)	24 (16.0%)	< 0.01
Stone-free rate	100%	96.7%	
Average burden score ± SD	7.8 ± 3.0	9.0 ± 3.5	< 0.01

## Discussion

This study aimed to quantify the burden of medical interventions by using a single score. The BS is the sum of all weights of the interventions, including all diagnostic and therapeutic measures, all complications, and ancillary procedures. Unlike previous scales, BS reflects the burden of all aspects of treatment from the patient’s perspective, a viewpoint often overlooked even in well-conducted prospective trials. These trials primarily focused on the outcomes and complications of the tested treatments and often neglected the burden of uncomplicated interventions. For example, in a randomized trial comparing thymectomy plus prednisone with prednisone alone in patients with myasthenia gravis, the investigators reported on the reduction in myasthenia score and the requirement for prednisone in the surgical group, but they overlooked the burden of median sternotomy [9]. In another randomized study comparing Nissen fundoplication with active medical treatment in patients with reflux-related heartburn, the measured outcomes were symptom scores and adverse events, while the burden of hospitalization and surgery was ignored [10].

The scale proposed in this study was tested in three clinical scenarios in urology, where guidelines offer two treatment options and there are no comparative randomized studies or clear-cut recommendations in the literature.

### Small renal masses

The groups in this cohort were not the same. Patients treated with PA were significantly older and sicker than those treated with PN, and their tumors were smaller by an average of 6 mm. The study confirmed an excellent prognosis with ≥ 97% 5-year recurrence-free survival after both PA and PN for all T1a tumors [11]. Complications were rare after both procedures; however, 13.2% of PA-treated patients required a second procedure compared to only 0.7% of patients treated with PN. The BS for PA was less than half that of PN (12.5 ± 6.4 vs. 27.3 ± 7.7, *p* < 0.01), and was lower even in patients who required a second PA. This difference is mainly due to the general anesthesia, laparoscopic surgery, and postoperative overnight observation required for PN, whereas PA is performed using moderate levels of conscious sedation, allowing for discharge after just a few hours of observation. Nevertheless, PA cannot be performed in tumors that are surrounded by the gut or near the ureter, whereas PN can be performed in almost any anatomical situation.

### T2-4a, N0M0 bladder cancer

This study showed an expected 3-year disease-specific survival of 73% after both RC and TMT, even though the TMT-treated patients were 5 years older (*p* < 0.01). A finding that is in accordance with current literature [12]. Patient selection is the key to success in this situation. For example, in one study, the 5-year disease-specific survival rate was 79.2% in cisplatin-eligible patients with tumors smaller than 3 cm compared to 33.9% in patients without one of these features [13]. Due to a lower mutilation, the BS of TMT was significantly lower than that of RC (average of 42.0 vs. 53.8, *p* < 0.01).

### Upper ureteral stones ≤ 10 mm

Patients with upper ureteral stones ≤ 10 mm who were treated with ureteroscopy were significantly older than those treated with SWL. Stone diameters were similar (average of 7.3 mm in both groups), and both treatments resulted in high success rates with rare complications. However, the BS of ureteroscopy was significantly lower (7.8 ± 3.0 vs. 9.0 ± 3.5, *p* < 0.01), mainly because of the high rate of ancillary procedures required to achieve a stone-free status following primary SWL treatment. Therefore, ureteroscopy is the preferred treatment for patients with upper ureteral stones measuring ≤ 10 mm.

This study had several limitations. It is retrospective, and therefore, patient allocation was not random, and selection bias can not be ruled out. The weights of different burdens were determined by a multidisciplinary committee, but they do not necessarily represent a universal truth. These weights can be modified by future committees, possibly including patients’ representatives. Furthermore, future investigations may also include differential weights according to age, gender or other patient’s features.

The economic burden of different treatments was not addressed in this study. However, because economic factors often align with burden weights (e.g., hospitalization days, number of CT scans, anesthesia, and surgery), it is reasonable to expect that the economic burden follows the same pattern as BS [14, 15].

## Conclusions

Patients treated with PA for small renal masses had similar recurrence-free survival rates as those treated with PN but with less than half the BS due to periprocedural burden. When technically feasible, PA should be considered as a good treatment option for patients with small renal masses. Patients treated with TMT achieved cure rates comparable to those of patients treated with RC, but the lower mutilation burden resulting in a lower BS. This treatment strategy should be considered for appropriately selected patients with T2-4a, N0, M0 bladder cancer. For patients with upper ureteral stones ≤ 10 mm, ureteroscopy and SWL offer similar success rates; however, due to the lower burden of ancillary procedures, ureteroscopy has a significantly lower BS and should be the preferred treatment.

BS is a tool for patient consultation when multiple treatment options are available. This concept can be implemented in other medical fields, with appropriate weights assigned to different interventions.

## Electronic supplementary material

Below is the link to the electronic supplementary material.

Supplementary Material 1

Supplementary Material 2

Supplementary Material 3

Supplementary Material 4

## Data Availability

No datasets were generated or analysed during the current study.
