# Supplementary material for: Quantifying treatment burden: the patient burden score a study of 758 patients across three clinical urologic scenarios
Source: World J Urol. 2024 Nov 27;42(1):650. doi: 10.1007/s00345-024-05378-3 (PMC11602774; doi:10.1007/s00345-024-05378-3)
Supplement: Supplementary file 1 — Supplementary Material 1 [file 345_2024_5378_MOESM1_ESM.docx]

**Supplementary Material 1: Burden of interventions in patients with small renal mass, bladder cancer (stages T2-4a, N0, M0), and upper ureteral stones ≤ 10 mm.**

| Event | Weight(points) |
| --- | --- |
| Expected Events |  |
| Daycare hospitalization (per day, up to four points) | 1 |
| Overnight hospitalization (per day, up to 14 points) | 2 |
| General anesthesia (mask) | 2 |
| Regional anesthesia | 2 |
| General anesthesia (tube) | 3 |
| Anesthesia related complications (prolonging hospitalization) | 3 |
| Outpatient clinic visit (up to four points) | 0.5 |
| Cystoscopy (stent removal) | 1 |
| Non-contract CT (or another non-contrast imaging, up to two points) | 1 |
| IV contrast enhanced CT (up to a maximum of four points) | 2 |
| Surgical or Oncological Procedures |  |
| Ureteroscopy | 2 |
| Shockwave lithotripsy | 2 |
| Transurethral resection of bladder tumor | 2 |
| Laparoscopy | 6 |
| Laparotomy | 8 |
| Trimodal therapy bladder | 10 |
| Adjuvant or neoadjuvant chemotherapy uncomplicated | 6 |
| Adjuvant or neoadjuvant chemotherapy complicated | 10 |
| Planned Organ Loss |  |
| Loss of a kidney (radical or partial, no need for replacement therapy) | 5 |
| Loss of a kidney (need for replacement therapy) | 40 |
| Loss of bladder (partial cystectomy, remaining part functional) | 6 |
| Loss of bladder with incontinent diversion | 20 |
| Loss of bladder with continent diversion | 10 |
| Pain Management |  |
| Pain managed enterally (each day, up to two points) | 0.5 |
| Pain managed parenterally (each day) | 1 |
| Further Surgery (after primary procedure failure) |  |
| Further surgery under anesthesia (in addition to its primary weight) | 2 |
| Unexpected Events (post-operative complications or post trimodal treatment) |  |
| Emergency room visit | 1 |
| Infection (non-febrile) | 0.5 |
| Infection (febrile) | 1 |
| Infection (septic) | 3 |
| Bleeding (no blood transfusion) | 0 |
| Bleeding (with blood transfusion) up to four points | 1 for each pc |
| Bleeding controlled by angiography | 2 |
| Bowel complications (conservatively treated) | 2 |
| Bowel complications (surgically treated) | 6 |
| Ureter stricture (conservatively treated) | 2 |
| Ureteral stricture (surgically treated) | 4 |
| Ureteral stricture with temporary nephrostomy | 1 |
| Ureteral stricture with permanent nephrostomy | 6 |
| End stage bladder | 20 |
| Deep vein thrombosis | 2 |
| Pulmonary embolism | 6 |
| Myocardial Infarction | 6 |
| cardiac arrhythmia managed medically | 2 |
| cardiac arrhythmia managed invasively | 4 |
| cerebrovascular event- no permanent neurological deficit | 4 |
| cerebrovascular event- permanent neurological deficit | 20 |
| hernia (conservatively treated) | 2 |
| hernia (surgically treated) | 4 |
| Nerve damage with no motoric disability | 2 |
| Nerve damage with motoric disability | 6 |
| Hematuria post radiation-conservatively treated | 1 |
| Hematuria post radiation-nephrostomy | 4 |
| Hematuria post radiation-surgically treated | 3 |
| Rectal bleeding post radiation- conservatively treated | 1 |
| Rectal bleeding post radiation- surgically treated | 3 |
| Post- chemotherapy diarrhea (conservatively treated) | 1 |
| Post- chemotherapy diarrhea (hospitalization) | 3 |
| Urinary incontinence (up to two pads a day) | 1 |
| Urinary incontinence (three or more pads a day) | 3 |
| Post treatment mortality | 100 |
